# *H. pylori* Eradication Treatment Alters Gut Microbiota and GLP-1 Secretion in Humans

**DOI:** 10.3390/jcm8040451

**Published:** 2019-04-04

**Authors:** Isabel Cornejo-Pareja, Gracia M. Martín-Núñez, M. Mar Roca-Rodríguez, Fernando Cardona, Leticia Coin-Aragüez, Lidia Sánchez-Alcoholado, Carolina Gutiérrez-Repiso, Araceli Muñoz-Garach, José C. Fernández-García, Isabel Moreno-Indias, Francisco J. Tinahones

**Affiliations:** 1 Department of Endocrinology and Nutrition, Virgen de la Victoria Hospital (IBIMA), Malaga University, 29010 Malaga, Spain; isabelmaria_cornejo@hotmail.com (I.C.-P.); graciamaria_mn@hotmail.com (G.M.M.-N.); fernandocardonadiaz@gmail.com (F.C.); leticia.coin@gmail.com (L.C.-A.); l.s.alcoholado@gmail.com (L.S.-A.); carogure@hotmail.com (C.G.-R.); aracelimugar@gmail.com (A.M.-G.); josecarlosfdezgarcia@hotmail.com (J.C.F.-G.); 2 Centro de Investigacion Biomédica en Red de la Fisiopatología de la Obesidad y Nutrición (CIBEROBN CB06/003), Instituto de Salud Carlos III, 28029 Madrid, Spain; 3 Department of Endocrinology and Nutrition, Puerta del Mar University Hospital, 11009 Cadiz, Spain; maroca80@gmail.com

**Keywords:** GLP-1 secretion, gut microbiota, antibiotic, *H. pylori*

## Abstract

Changes in the intestinal microbial community and some metabolic disturbances, including obesity and type2 diabetes, are related. Glucagon-like peptide-1 (GLP-1) regulates glucose homeostasis. Microbiota have been linked to incretin secretion. Antibiotic use causes changes in microbial diversity and composition. Our aim was to evaluate the relationship between microbiota changes and GLP-1 secretion. A prospective case-control study with a *Helicobacter pylori*-positive patient model involving subjects under eradication therapy (omeprazole, clarithromycin, and amoxicillin). Forty patients with *H. pylori* infection and 20 matched participants, but negative for *H. pylori* antigen. Patients were evaluated before and two months after treatment. We analyzed anthropometric measurements, carbohydrate metabolism, lipid profile, and C-reactive protein. Gut microbiota composition was analyzed through 16S rRNA amplicon sequencing (IlluminaMiSeq). Eradication treatment for *H. pylori* decreased bacterial richness (Chao1, *p =* 0.041). Changes in gut microbiota profiles were observed at phylum, family, genus and species levels. GLP-1 secretion and variables of carbohydrate metabolism were improved. Correlations were seen between GLP-1 changes and variations within microbial community abundances, specifically *Bifidobacterium adolescentis*, the *Lachnobacterium* genus, and *Coriobacteriaceae* family. A conventional treatment to eradicate *H. pylori* could improve carbohydrate metabolism possibly in relation with an increase in GLP-1 secretion. GLP-1 secretion may be related to alterations in intestinal microbiota, specifically *Lachnobacterium, B. adolescentis* and *Coriobacteriaceae*.

## 1. Introduction

The discovery of the great impact of intestinal microbiota on metabolic disease has led to its intensive study in recent decades and suggests a key role in the regulation of adiposity and host energy and carbohydrate homeostasis [1]. Despite the relative stability of the microbiota in adults, it varies between groups of humans and even from person to person due to specific or intrinsic differences such as age, host genome [2], and health status, as well as extrinsic factors such as diet, use of prebiotics and probiotics, stress, use of antibiotics or other drugs, and environmental factors such as temperature, climate, or environmental pollution [3,4]. The intestinal microbiome in humans is considered a virtual externalized, metabolically adaptable, flexible, and rapidly renewable organ, involved in the maintenance of host homeostasis and metabolism; consequently, its composition and modifications are of special interest. An increasing number of studies show that changes in certain intestinal bacterial populations are associated with the development of diseases such as obesity and type 2 diabetes [5,6]. In particular, the two most abundant phyla of the human intestinal microbiota, Firmicutes and Bacteroidetes, are enriched with genes that encode multiple enzymes involved in carbohydrate and lipid metabolism [6]. 

Glucagon-like peptide-1 (GLP-1) is a 30-amino acid incretin polypeptide produced in the enteroendocrine L cells of the intestinal mucous membrane from proglucagon, mainly in the distal small intestine (ileum) and colon. GLP-1 regulates glucose homeostasis [7]. GLP-1 has trophic effects on pancreatic β cells [8,9], reduces acid secretion in the stomach, and delays gastric emptying. GLP-1 also regulates appetite by inducing a feeling of satiety. 

Microbiota has been linked to the secretion of incretins. Specific bacteria—including *Bifidobacterium*, *Lactobacillus*, *Lactobacillus reuteri*, and *Akkermansia muciniphila* [10]—contribute to the modulation of incretin hormones such as GLP-1. Numerous recently published studies in both humans [11] and rodents [12] have analyzed the association between the changes in the intestinal microbial community induced by increased dietary fiber with the differential production of short-chain fatty acids (SCFAs)—primarily butyrate, propionate, and acetate—and the release of gastrointestinal hormones GLP-1 and PYY (peptide YY), involved in metabolic health and protecting against obesity and diabetes, although the underlying mechanisms are incompletely understood [11,12].

We have previously found that an antibiotic therapy was able to disturb gut microbiota population, and more importantly, these changes were related to an improvement of the glucose metabolism [13]. It is widely accepted that the bacteria in our gut can modulate intestinal enteroendocrine L cell function, thereby modifying the release of GLP-1 [10,14]. Although knowledge of the underlying molecular mechanisms is limited, attention is focused on how the metabolites produced by the intestinal microbiota—especially SCFAs (degradation products of fermentable dietary fiber), oleoylethanolamide (a bioactive lipid compound related to the endocannabinoid system), and secondary biliary acids (deoxycholic and lithocholic acids)—activate G protein coupled receptors expressed in the plasma membrane of L cells GP41 and GP43, GP119, and TGR5, respectively, regulating the release of GLP-1 in the intestine, among other functions [10,15]. In addition, it has recently been reported that the expression of these receptors may be affected by the composition of the intestinal microbiota through epigenetic regulation of gene expression [16].

Accordingly, our main objective is to verify the relationship between changes in microbiota and GLP-1 secretion. To achieve this objective, we propose to study a population of patients who receives antibiotic treatment for eradication of *H. pylori* to demonstrate whether the foreseeable changes in the microbiota produced by the antibiotic treatment are able to modify GLP-1 secretion.

## 2. Materials and Methods

### 2.1. Study Design and Population

Forty consecutive *H. pylori*-infected adults were recruited and selected from the Department of Microbiology through detection of *H. pylori*-positive antigens by immunochromatography, between 2015–2016. Sample size was assessed considering a reduction in richness of 16% because of the antibiotic therapy based on previous microbiota studies [17] and a pilot study The sample size totaled 35 subjects for the intervention study. Inclusion criteria were: age 18–65 years, and first infection with *H. pylori*. In addition, a control group of 20 non-infected participants matched for age, anthropometry, sex, and dyspeptic symptoms, with a negative result for *H. pylori* antigen, was also selected from the Department of Microbiology and recruited those with similar clinical characteristics. Exclusion criteria were: diagnosis of type 1 or type 2 diabetes, documented previous treatment for *H. pylori*, use of antibiotics within 3 months prior to the start of the study, consumption of probiotics, individuals previously having undergone bariatric or gastric surgery, and non-acceptance of informed consent. Overall, 97.5% of subjects managed to eradicate the bacteria adequately, being confirmed through the antigenic determination in the stool test. The patient who did not eradicate *H. pylori* infection was excluded from study, because they had not completed antibiotic treatment satisfactorily.

This prospective intervention study included two visits for the patients, who were assessed prior to and two months after eradication treatment (omeprazole 20 mg, clarithromycin 500 mg, amoxicillin 1000 mg twice daily for 10 days), and only one visit for the control group. All visits included a physical examination, fasting blood sample, and a 75-g oral glucose tolerance test (OGTT) with determinations at 30, 60, and 120 min after baseline. 

The study protocol was reviewed and approved by the Medical Ethics Committee of Virgen de la Victoria University Hospital and conducted in accordance with the Declaration of Helsinki. All participants gave their written informed consent and were also verbally informed of the characteristics of the study.

Plasma glucose (mg/dL) was analyzed using the Modular DPD biochemical system (Roche Diagnostics, Risch-Rotkreuz, Switzerland). Glycosylated hemoglobin (HbA1c, in %) was measured using an immunoturbidimetric method for completely hemolyzed anticoagulated blood (COBAS INTEGRA 700 autoanalyzer, Roche Diagnostics, Risch-Rotkreuz, Switzerland). Total cholesterol (mg/dL), triglycerides (mmol/L), low-density lipoprotein cholesterol (mg/dL) and high-density lipoprotein cholesterol (mg/dL), were quantified by enzymatic colorimetry using the Modular DPD biochemical autoanalyzer (Roche Diagnostics, Risch-Rotkreuz, Switzerland).

For GLP-1 determination, plasma was collected in 3 mL Vacuette^®^ tubes (K3E K3EDTA, Greiner bio-one, Kremsmünster, Austria) and was treated by the addition of 50 μL of aprotinin (Protease enzyme inhibitor). The samples were immediately centrifugated for 15 min at 4000 rpm. The resulting plasma was frozen at −80 °C until analysis. Plasma GLP-1 was measured manually using commercial kits (human GLP-1 EIA Kit, GEN-TAUR Belgium, Kampenhout, Belgium) and expressed in ng/mL.

Stool samples were collected at each visit and immediately frozen at −80 °C until DNA extraction. Detection of *H. pylori* antigen in feces by immunochromatography (LETI Laboratories) was used to confirm eradication after antibiotic treatment.

Briefly, the procedure for microbiota analysis was as following: Stool DNA was extracted from stool samples using the QIAamp DNA Stool Mini Kit, according to the manufacturer’s protocols (Qiagen, Germany). Stool DNA concentrations were measured using a Qubit^®^ (London, Great Britain) Fluorometric (ThermoFisher Scientific, Waltham, MA, USA). The fecal bacterial microbiota composition was determined using tag-encoded16S rRNA gene Miseq-based (Illumina, San Diego, CA, USA) high-throughput sequencing. The 16S rRNA V3-V4 amplicon (amplicon size ~460bp) was amplified by polymerase chain reaction (PCR). A bioanalyzer (Agilent 2100, Agilent Technologies, Santa Clara, CA, USA) was used to verify the size of the PCR product. AMPure XP beads (Beckman Coulter Genomic, Morrisville, CA, USA) were used to purify the free primers and primer dimer species in the amplicon product. Dual indices and Illumina sequencing adapters were attached to sequence the amplicons, using the Nextera XT Index Kit (Illumina, San Diego, CA, USA). Paired-end sequencing of amplicons was conducted on the IlluminaMiSeq platform using the v3 kit generating 2 × 301 nucleotide reads (Illumina, San Diego, CA, USA).

Food intake was evaluated by using seven 24-h dietary recalls for case and control groups, and total energy (Kcal/day), macronutrients like carbohydrates, proteins, fats (g/day), and micronutrients (mg/day) were analyzed. The energy and nutrient intake were calculated using DIAL software (Alce Ingeniería, 2008). 

### 2.2. Bioinformatic Analysis

The merged paired-end reads were analyzed using the Quantitative Insights Into Microbial Ecology (QIIME) tool (version 1.9.1, open source software). The operational taxonomic units (OTUs) were generated by clustering sequences with 97% similarity and the representative sequences, selected as the most abundant in each cluster, underwent taxonomic alignment by UCLUST consensus to obtain the taxonomic assignment and relative abundance of each OTU using the Greengenes 16S rRNA gene database. Alpha diversity analyses (microbial diversity within samples) were performed using QIIME. The level of alpha diversity was calculated using the alpha rarefaction workflow, establishing a threshold of 34,385 sequences, corresponding to the 85% of the sample with the lowest representativeness. Rarefied data were used for the downstream analysis. Moreover, in order to increase the statistical power, OTUS that were not found in at least five different samples were excluded from the analysis. These sequences were assigned to 12 phyla, 49 families, 75 genera, and 42 different species. Alpha diversity was estimated using the Chao1 and Shannon indices, as well as the observed species. Raw data can be found in the SRA database public repository from NCBI within the BioProject accession number PRJNA517270. Additionally, PICRUSt analysis was used to predict metagenome function by picking operational taxonomic units (OTUs) against the Greengenes database [18].

### 2.3. Statistical Analysis

Statistical analysis of the data was performed primarily using the SPSS program (version 22.0.0 for Windows, SPSS Iberica, Madrid, Spain), while QIIME (version 1.9.1, open source software) was used to complete the evaluation of microbial communities in the study groups. STAMP software was used for the analysis of PICRUSt results [19]. Measurements of central tendency (mean) and dispersion (standard error of the mean) were used for continuous variables. The Kolmogorov–Smirnov test was used to assess normality.

Hypothesis testing of the means for the variables of interest was performed using the Student’s *t*-test between two independent comparison groups and the Student’s *t*-test for paired samples to compare the study group before and after the antibiotic eradication treatment. Non-parametric variables were evaluated by Mann–Whitney and Wilcoxon signed-rank tests. The correlation between quantitative variables including analytical, clinical, and microbial populations was analyzed using the Spearman bivariate correlations test. The area under the curve (AUC) was calculated using the trapezoidal rule (Riemann summation). Statistical significance was established at *p* < 0.05. *p* values were corrected for multiple comparisons using the Benjamini–Hochberg method when appropriate.

## 3. Results

### 3.1. Carbohydrate Metabolism

The clinical and anthropometric characteristics of the study groups are presented in Table 1. No significant changes were found after antibiotic eradication treatment. No differences were found in C-reactive protein, triglyceride, or total cholesterol levels after the eradication therapy. However, there was a significant decline in HbA1c levels following antibiotic therapy (*p =* 0.005). In relation to lipid metabolism, high-density lipoprotein cholesterol levels increased significantly after antibiotic therapy (*p =* 0.021). Low-density lipoprotein cholesterol levels were lower in healthy individuals than in *H. pylori*-infected subjects before antibiotic treatment (*p =* 0.036). No statistically significant between patients and control group differences were observed in the dietary intake of energy, and macro or micronutrients, as well as dietary fiber (*p* > 0.05) (data not shown).

**Figure 1:**
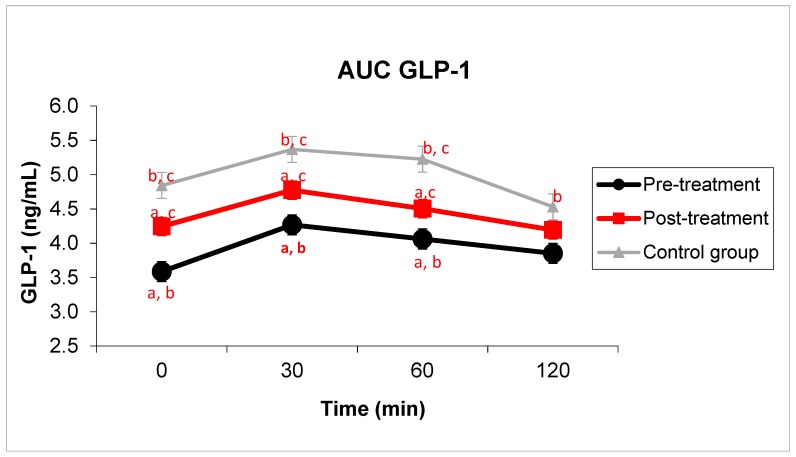
Changes in Glucagon-like peptide-1 (GLP-1) levels after antibiotic therapy compared to the control group. GLP-1 levels in *H. pylori*-infected patients at baseline and after 75g-OGTT and after 2 months of post-antibiotic treatment compared to the control group. Student’s *t*-test for paired samples and Student’s *t*-test between independent samples were used to compare the group of *H. pylori*-infected patients before and after antibiotic treatment, and control group, respectively. Equal letter means differences between groups. ^a^: differences between *H. pylori*-infected individuals before and after antibiotic treatment, *p* < 0.05. ^b^: differences between *H. pylori*-infected patients and the control group, *p* < 0.05. ^c^: differences in patients undergoing antibiotic therapy compared to the control group, *p* < 0.05.

In relation to GLP-1 levels, significantly higher mean GLP-1 levels were found in healthy individuals, both at baseline (4.8 ± 0.4 ng/mL vs. 3.6 ± 0.3 ng/mL; *p =* 0.002), and at minutes 30 (5.4 ± 0.4 ng/mL vs. 4.27 ± 0.36 ng/mL; *p =* 0.007), 60 (5.2 ± 0.3 ng/mL vs. 4.06 ± 0.36 ng/mL; *p =* 0.002), 120 (4.7 ± 0.4 ng/mL vs. 3.85 ± 0.31 ng/mL; *p =* 0.025) and in the area under the GLP-1 curve (609 ± 40.2 ng·min/mL vs. 479.1 ± 39.5 ng·min/mL; *p =* 0.004) with respect to patients infected with *H. pylori*. In Figure 1, we also found that 2 months after the antibiotic eradication treatment, GLP-1 levels in *H. pylori*-positive subjects showed increased values, nearing those of the control group at baseline (4.2 ± 0.4 ng/mL vs. 3.6 ± 0.3 ng/mL; *p* < 0.001). We also found a rise in GLP-1 levels after OGTT at minutes 30 (4.77 ± 0.43 ng/mL vs. 4.27 ± 0.36 ng/mL; *p =* 0.013), at minute 60 (4.51 ± 0.42 ng/mL vs. 4.06 ± 0.36 ng/mL; *p =* 0.007), and in the area under the GLP-1 curve (535.5 ± 47.7 ng·min/mL vs. 479.1 ± 39.5 ng·min/mL; 0.002). Although without statistical significance, a tendency was found at minute 120 (4.19 ± 0.38 ng/mL vs. 3.85 ± 0.31 ng/mL; *p =* 0.085).

These changes in GLP-1 levels following antibiotic treatment occurred in parallel with improvements in the carbohydrate metabolism of *H. pylori*-infected subjects, with decreases in HbA1c levels (5.5 ± 0.08% vs. 5.29 ± 0.06%, *p =* 0.005) and in the area under the glucose curve (16,684 ± 727 mg·min/dL vs. 15,894 ± 735 mg·min/dL; *p =* 0.006), as we have recently published [13].

### 3.2. Gut Microbiota

We used an otherwise healthy *H. pylori*-positive patient model receiving antibiotics for a limited period of time. This enabled us to assess the repercussions of antibiotic use on intestinal microbiota and incretin hormone secretion over a short period of time, which prevented confounding factors derived from changes in weight, the presence of other diseases, concomitant use of other therapies, as well as ethical problems.

**Figure 2:**
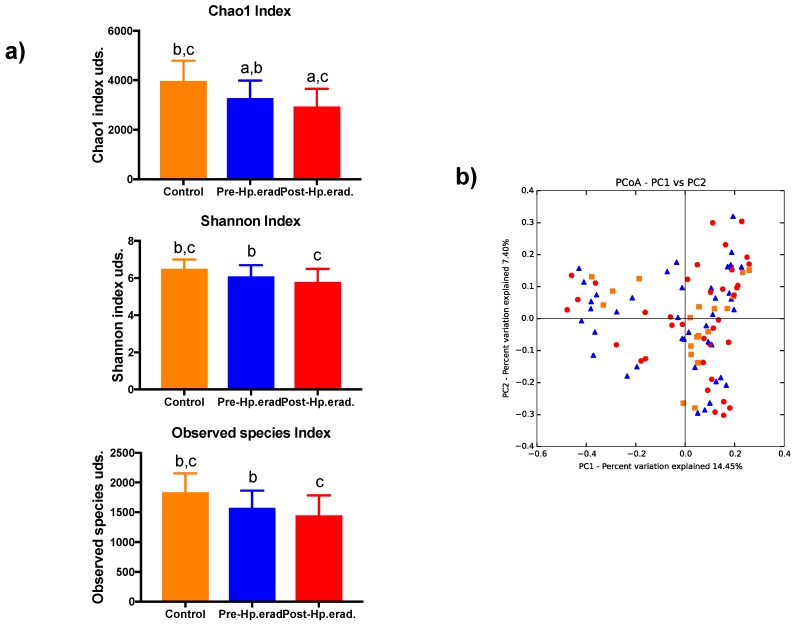
Estimation of diversity in control subjects and *H. pylori*-infected patients after antibiotic treatment. (**a**) Richness and diversity indices among different groups were compared. Wilcoxon test was used to compare the group of *H. pylori*-infected patients before and after antibiotic treatment. Equal letters indicate statistical differences between those groups. ^a^: differences between *H. pylori*-infected patients before and after antibiotic treatment, *p* < 0.05. ^b^: differences between *H. pylori*-infected patients compared to the control group, *p* < 0.05. ^c^: differences between patients’ post-antibiotic treatment versus control group, *p* < 0.05. All values are means ± standard error of the mean. (**b**) Clustering of gut microbiota populations according to the study groups by principal coordinate analysis (PCoA) using the Bray–Curtis dissimilarity. No statistical differences were observed between groups. Orange squares belong to the control subject group; blue triangles to patients before treatment; and red dots to patients after treatment.

We found that the antibiotic treatment used to eradicate *H. pylori* affected the diversity of the intestinal microbiota. We examined the alpha diversity or intrinsic diversity through the rarefaction curves estimated by the Chao1, Shannon, and observed species indexes, revealing clear differences between the groups studied. The control group subjects showed greater diversity and richness, with statistically significant differences compared to the *H. pylori*-infected individuals, both before and after antibiotic treatment. In the *H. pylori*-infected patients, the treatment clearly affected richness (*p =* 0.041), which indicates a decrease in the number of bacteria recorded. Figure 2. Besides, the overall changes occurred in the gut microbiota populations were assessed with the estimation of the beta diversity through the Bray–Curtis similarity index. Figure 2 shows no statistically differential between control subjects and *H. pylori*-infected patients before and after the eradication therapy (*p =* 0.113). However, if phylogenetic information is added with the Unifrac distance, significant differences were observed between control subjects and *H. pylori* infected patients as well as after the antibiotic treatment (*p* < 0.05) (see [13]).

Concerning the relative abundance of each bacterium in the fecal samples collected, the dominant bacterial phyla within the study groups were Firmicutes and Bacteroidetes. Actinobacteria, Proteobacteria, and Verrucomicrobia contributed with smaller proportions, between 1–5%. With respect to the phyla Bacteroidetes and Firmicutes, significant differences were found between patients infected with *H. pylori* both before and after antibiotic treatment compared to the control group.

In addition, a decrease in the relative abundance of Actinobacteria was found in the patients after antibiotic treatment compared to both baseline (0.27 ± 0.07% vs. 0.78 ± 0.15%, *p* < 0.001) and the control group (0.27 ± 0.07% vs. 0.97 ± 0.27%, *p* < 0.001). There was also a decrease in relative abundance in the phylum Tenericutes after antibiotic therapy compared to baseline (0.02 ± 0.01% vs. 0.05 ± 0.02%, *p =* 0.043), approaching the levels of the control group (0.02 ± 0.01% vs. 0.01 ± 0.00%, *p =* 0.037). The use of antibiotic treatment in *H. pylori*-infected patients resulted in important changes in the intestinal microbiota in phyla, families, genera, and species. A detailed description of the changes in the intestinal microbiota after antibiotic treatment has been previously found by our group [13].

**Figure 3:**
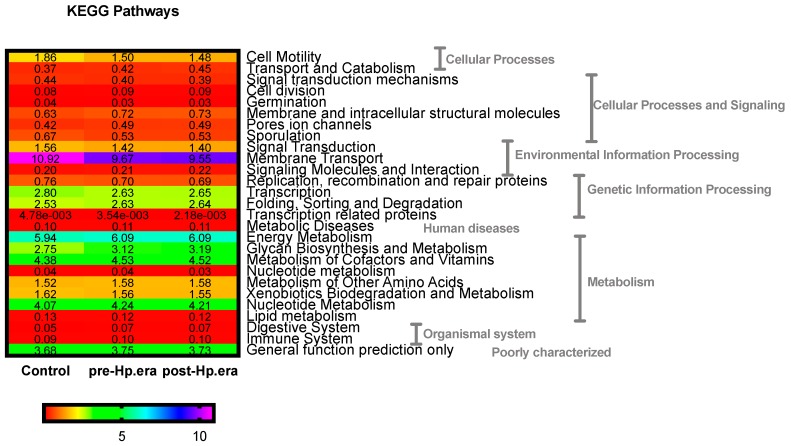
Predicted functional composition of metagenomes based on 16S rRNA gene sequencing data of *H. pylori* infected patients and after the eradication therapy and healthy control subjects. Heatmap shows the differentially abundant Kyoto Encyclopedia of Genes and Genomes (KEGG) pathways identified in the three study groups (FDR-ajusted-P ≤ 0.05).

On the other hand, we performed a PICRUSt analysis in order to predict the metagenome function. Figure 3 shows that cellular processes and signaling, environmental and genetic information processing, human diseases, metabolism, organismal system, and poorly characterized pathways were found to be statistically different between subjects (FDR-adjusted-P < 0.05). Because of their highest abundance, especially relevant pathways seem to be the differences found between control and *H. pylori* infected patients pre- and post-eradication treatment in environmental information processing where control subjects presented a higher capacity, which is especially relevant in membrane transport (FDR-adjusted-P = 0.003), energy metabolism (FDR-adjusted-P = 0.004), metabolism of cofactors and vitamins (FDR-adjusted-P = 0.008), and nucleotide metabolism (FDR-adjusted-P = 0.022).

Furthermore, when we related this changes in the microbiota population to GLP-1 secretion we found that some families, genera, and species of the phyla Actinobacteria and Firmicutes showed positive correlations with GLP-1 levels, while families and genera of the phylum Bacteroidetes and the species *Blautia producta* belonging to the phylum Firmicutes showed negative correlations with GLP-1 levels. These correlations were found in *H. pylori*-infected patients pre- and post-antibiotic treatment, as well as in the control group. In the control group, specifically, we noted that the genus *Megamonas* (a genus that has been related to carbohydrate metabolism [20]), showed positive correlations with baseline GLP-1 levels, the area under the GLP-1 curve, and at all the times tested after OGTT. Most of the correlations found between the different groups of bacteria and GLP-1 levels were observed 60 min after OGTT, with positive correlations for families and genera of the phylum Firmicutes and negative correlations for the phylum Bacteroidetes (Table S1).

**Figure 4:**
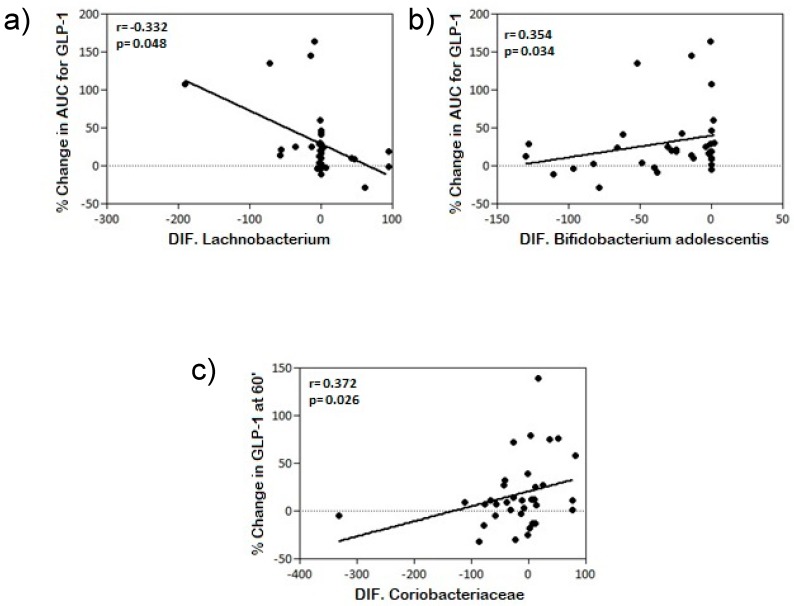
Correlation between changes in GLP-1 levels at minute 60 and the AUC for GLP-1 with percentage change in microbial community after antibiotic treatment: (**a**) with *Lachnobacterium* percentage change; (**b**) with *Bifidobacterium adolescentis* percentage change; and (**c**) with *Coriobacteriaceae* percentage change.

In *H. pylori*-infected individuals (pre-antibiotic treatment), GPL-1 levels at minute 60 and the area under the GLP-1 curve showed the highest correlations with the microbial populations. Families, genera and species of the phylum Proteobacteria showed positive correlations with GLP-1 levels 60 min after OGTT and in the area under the GLP-1 curve only in the *H. pylori*-infected group prior to eradication treatment. The species *Blautia producta* showed negative correlations with GLP-1 levels at all the time points except 120 min after OGTT (Table S2). Following antibiotic eradication treatment, correlations with the phylum Proteobacteria disappeared, and the species *Bifidobacterium longum* and the genus *Prevotella* showed the greatest correlations, positive and negative respectively, with GLP-1 levels (Table S3). Of particular interest were the correlations between changes in GLP-1 levels with the disturbances experienced by different families, genera, and species of the microbiota in *H. pylori*-infected subjects after antibiotic treatment. Our findings show that changes in the area under the GLP-1 curve correlated positively with changes in bacterial communities of the species *Bifidobacterium adolescentis*, phylum Actinobacteria, and negatively with the genus *Lachnobacterium*, phylum Firmicutes, while changes in the microbial community from the *Coriobacteriaceae* family, phylum Actinobacteria, correlated positively with changes in GLP-1 levels at 60 min after an OGTT. Table 2, Figure 4, and supplementary Figure S1 show the relative abundances of these members of the microbial community.

Spearman correlation test was used to compare the microbial differential abundance respect to GLP-1 changes in *H. pylori*-infected patients after antibiotic treatment.

## 4. Discussion

Few human interventional studies have investigated the effect of antibiotic treatment on gut hormone secretion or glucose metabolism. We report that a broad-spectrum antibiotic therapy in otherwise healthy *H. pylori* positive patients causes an increase of incretin hormones (GLP-1) accompanied by an improvement in carbohydrates metabolism variables.

GLP-1 is one of the most potent stimulators of insulin secretion. This secretion is regulated by different signaling pathways, the main one through the direct stimulation due to the contact of nutrients with the apical membrane of the enteroendocrine L cells of the distal small intestine. The rise in GLP-1 levels following meal ingestion cause increased postprandial insulin secretion and improved insulin sensitivity in peripheral tissues [21]. In this study, we found that 2 months after the completion of antibiotic therapy there was an overall increase in GLP-1 levels, with values approaching those of the control group, which can be related to improved carbohydrate metabolism, particularly with lower HbA1c levels. We have observed a decrease of 0.21% in HbA1c after antibiotic treatment in non-diabetic subjects, a valuable effect for the average HbA1c levels by age (5.19–5.37%) that could intensify/be magnified in diabetic patients [22].

Numerous studies are aimed at clarifying how the intestinal microbiota intervenes in the host metabolism by regulating the secretion of incretin hormones such as GLP-1. Enteroendocrine cells in the gut are distributed throughout the epithelial lining and have been identified as those involved in communication between the microbiota and the host through bacterial metabolite signaling [10,23]. The signaling pathways of the intestinal microbiota, through the production of SCFAs, secondary bile acids, and indole, among other metabolites, are well accepted. Many studies [5,24,25] have explored how modulation of the microbiota plays an important role in carbohydrate metabolism, with improved glycemic control and insulin sensitivity, which can occur through multiple pathways including modulation of incretin hormones by the microbiota. We further explored this latter aspect through the development of our study.

Although the microbiota is generally stable within individuals, over time its composition may be altered due to external disturbances. A multitude of extrinsic factors could be listed that can have a major influence on changes in the intestinal microbiota but one of the most important etiological factors involved is the use of antibiotics, with important changes in intestinal microbiota seen following their use [26,27]. Several studies have explored this field in both animals and humans, concurring on changes in the intestinal microbiota over a period of time, with their subsequent re-establishment [27,28], while others have described persistent effects with a long-term impact on the microbiota, where recovery may sometimes be incomplete [29,30,31], causing rapid and significant reductions in taxonomic richness, diversity, and uniformity [29,32]. In agreement with the literature [26,31,33,34], we observed early secondary changes in biodiversity after conventional antibiotic therapy [32,34]. However, Bray–Curtis dissimilarity showed that microbiota population was not significantly changed with the antibiotic treatment, although Unifrac distance indicated the contrary [13], indicating that the phylogenetic associations among the gut microbiota members is an important factor for the development of the gut microbiota functionality. In this manner, PICRUSt analysis showed that important pathways of metabolism and the processing of the environmental information were affected with the treatment.

**Figure 5:**
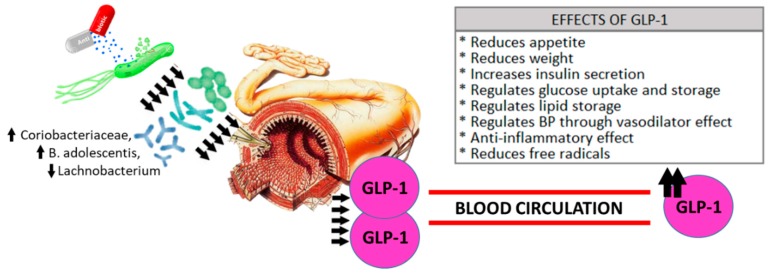
Microbiota and GLP-1 secretion. The use of conventional antibiotic treatment generates modifications in the intestinal microbiota. In this study, these changes in specific microbial communities (*Coriobacteriaceae*, *B*. *adolescentis*, *Lachnobacterium*) have been related with an increase the secretion of GLP-1 and its beneficial effects.

Declines in diversity, usually named dysbiosis, have been commonly associated a deterioration in health status [5,24,35,36]. Similarly, the presence of low bacterial richness has been associated with increased overall adiposity, insulin resistance, dyslipidemia, and a more pronounced inflammatory phenotype [37,38]. Indeed, we have observed a decline in the diversity of the *H. pylori*-infected patients even before the eradication treatment. Gao et al. did not find statistically significant differences in the alpha-diversity of *H. pylori*-infected and non-infected patients, although in a smaller sample size [39]. However, *H. pylori* infection could impair the transmission of oral bacteria to the gut [40]. It is well-known that *H. pylori* infection produces inflammation in the gastric mucosa [41]. In addition, it has been shown in animal studies that *H. pylori* infection could interfere in the metabolism of the host, impairing several energy modulating hormones [42]. Taken these results together, we hypothesize that both the inflammation of the gastric mucosa and the alteration in host metabolism could impair GLP-1 secretion and could be one of the reasons for lower concentrations of baseline GLP-1 in *H. pylori*-infected patients something that could be also the reason of a less secretion of GLP-1 in these patients, as well as the decline in the microbial diversity of these patients. Thus, these facts deserve further investigation. However, a high diversity is not necessarily ‘better’ or ‘healthy’. Diversity is the starting point to investigate the ecological mechanisms within the whole microbiota population [43]. In addition, microbial alterations induced by the use of antibiotic therapy affect bile acid metabolism and insulin sensitivity, both in humans and in animal models with rodents [44]. However, other studies [45] have demonstrated beneficial effects of antibiotic treatment on metabolic alterations in obese mice, leading to improvements in carbohydrate intolerance, decreased metabolic endotoxemia, and markers of inflammation and oxidative stress, associated with reduced microbial diversity. In line with this research, in our study, despite finding a lower diversity and microbial richness after the use of antibiotic therapy, parallel beneficial changes were produced in carbohydrate homeostasis with a decrease in HbA1c, a more favorable lipid profile with an increase in high-density lipoprotein cholesterol levels, and an increase in the concentration of hormones, such as GLP-1, regulating food intake; thus a decline in biodiversity does not necessarily imply a negative impact on carbohydrate metabolism as has been historically thought. In 2014, Vrieze et al., did not find changes in postprandial glucose tolerance or secretion of incretin hormones in obese patients with metabolic syndrome treated with vancomycin or amoxicillin [44]. Mikkelsen et al., found acute reductions in the abundance of gut bacteria but not objective modifications in glucose tolerance or release of gastrin, CCK, GIP (gastric inhibitory polypeptide), and GLP-1 after a short-term broad-spectrum antibiotic course (vancomycin, gentamycin, and meropenem) in healthy young males [46]. In our study, we have examined the effects of an antimicrobial therapy on incretin hormone secretion in humans as well as we have explored the possibility of a direct relationship between changes in the commensal microbial community and modulation of GLP-1 levels. Indeed, changes in GLP-1 levels could be related both to specific bacteria in the microbiota and to the use of antibiotics as seen in Figure 5.

The impact of the use of antibiotic treatment with amoxicillin and clarithromycin, as well as omeprazole, on GLP-1 secretion due to the direct disturbance that these drugs produce in certain genera and species of the human intestinal microbial community is an area of interest about which there is only limited knowledge at present. In this study, we found that—after the eradication treatment—some families, genera, and species of the phyla Actinobacteria and Firmicutes showed positive correlations with GLP-1 levels, whereas families and genera of the phylum Bacteroidetes and the species *Blautia producta* of the phylum Firmicutes showed negative correlations with GLP-1 levels. As far as we are aware, these are the first published results in humans.

Recent research has explored this issue in rodents. Hwang I et al. [47] analyzed how the use of antibiotic therapy (vancomycin and bacitracin) generates changes in the microbiota of obese rodents, finding a decrease in the proportion of Firmicutes and Bacteroidetes and an increase in Proteobacteria, which has been linked to improved systemic glucose intolerance and insulin resistance, through increased secretion of GLP-1. Rajpal DK et al. [48], however, determined that the use of narrow-spectrum antibiotics had different effects, possibly due to the different microbial changes they generated. While vancomycin (directed against Gram-positive bacteria) did not improve glycemic control or produce changes in GLP-1 levels, the use of ceftazidime (directed against Gram-negative bacteria) achieved a more favorable glycemic profile regarding insulin, glucose, and GLP-1 levels. These findings could be related to the changes in intestinal microbiota which showed higher proportions of Gram-positive bacteria, Firmicutes, and lower abundance of Gram-negative bacteria, especially Proteobacteria. Our study contributes further knowledge in this area by exploring the possible effects of a microbial community on the release of GLP-1, suggesting that specific changes in intestinal microbial composition generated by the direct effect of antibiotic treatment could be associated with overall increases in GLP-1 levels, both at baseline and after OGTT. Although not analyzed in our research, studies in rodents have explored how a decrease in the diversity of intestinal microbiota can favor resistance to GLP-1 [49] through a reciprocal interaction between microbiota and GLP-1 [50]. Grasset et al. [49] clearly showed that the decrease in the microbial diversity reduced the expression of GLP-1 receptors and hindered the production of GLP-1-induced nitric oxide, preventing activation of the intestine–brain axis to control insulin secretion and gastric emptying. This set of data supports the notion that eubiotic intestinal microbiota improves GLP-1 sensitivity, while an alteration in the bacterial population reduces it. Nonetheless, future studies are needed to confirm these observations of the most important bacterial groups involved in GLP-1 signaling and resistance.

We also found correlations between changes in the area under the GLP-1 curve after completion of the antibiotic treatment with amoxicillin and clarithromycin. These correlations were negative with the changes in the genus *Lachnobacterium* and positive with the changes in the species *Bifidobacterium adolescentis*, as well as between the variation in GLP-1 at 60 min after OGTT and the changes in the family *Coriobacteriaceae*.

Within the genus *Lachnobacterium*, some of its species such as *L. bovis* are able to ferment glucose, generating lactic acid, acetic acid, and butyric acid (SCFAs) as the main degradation products [51]. Butyric acid is one of the most abundant and important SCFAs in the gut due to its multiple effects, among which is its participation in energy homeostasis increasing the sensation of satiety and regulating appetite and energy intake [52], such as in carbohydrate homeostasis, favoring the secretion of GLP-1 and PYY in L cells, which can influence blood glucose and insulin response with improved insulin resistance [53,54]. Butyric acid is also believed to have anti-inflammatory properties and to reduce intestinal permeability with decreased endotoxemia [55,56]. Yadav et al. [53] demonstrated that modulation of the composition of intestinal microbiota through the use of probiotics stimulates the production of butyrate, which favors the secretion of GLP-1 in L cells, improving inflammatory status and insulin resistance.

*Bifidobacterium adolescentis* has the ability to degrade carbohydrates due to its α- and β-galactosidase and β-fructofuranosidase [57] activity and its ability to use SCFAs as an acetate for the production of butyrate [58]. Increases in the production of GLP-1 and intestinal peptide YY have also been described in relation to the increase of *Bifidobacterium* due to the use of some prebiotics. The increase in *Bifidobacterium* modulates inflammation in obese mice, increasing GLP-1 production and reducing intestinal permeability with positive effects on carbohydrate metabolism, favoring a decrease in insulin resistance and improving pancreatic β cell function [59,60]. In addition, studies on the microbiota of patients with type 2 diabetes have shown smaller amounts of *B. adolescentis* [61].

*Coriobacteriaceae* are widespread constituents of the human intestinal microbiota and have important interactions with the host. Some of its members are involved in bile acid metabolism [62], which appears to have an important insulin-sensitizing role in glucose homeostasis [63,64]. Unconjugated primary bile acids are metabolized by these bacteria into secondary bile acids that bind to TGR5 receptors, increasing energy expenditure in the muscle and favoring the secretion of GLP-1 in L cells [64]. In addition, a recent study of women with type 2 diabetes reported a decrease in their stool samples of the family *Coriobacteriaceae* [5]. Within this family, the genus *Collinsella* has been associated with insulin levels [65], and in the species *Collinsella aerofaciens* a gene has been identified that encodes a dehydrogenase enzyme capable of catalyzing both the oxidation of ursodeoxycholic acid to form 7-keto-lithocholic acid and the reduction of 7-keto-lithocholic acid to form deoxycholic acid, this enzyme being a useful biocatalyst for producing deoxycholic acid from precursors under appropriate conditions [66]. In both animal and human studies [67], ursodeoxycholic acid has been shown to have antioxidant and anti-inflammatory activity, improving insulin sensitivity and consequently glycemic homeostasis.

We found a possible limitation in our study, as *H. pylori* eradication could intervene in some way in GLP-1 secretion and therefore be a confusing factor. However, the strength of the current study is the fact that it was performed in otherwise healthy patients without confusing variables. Next step could be an antibiotic trial in patients without *H. pylori* infection, although an ethical problem could emerge. The current results could serve as the starting point of this new clinical trial. On the other hand, we have analyzed colonic microbiota through stool samples. GLP-1 secretion is mainly secreted in the proximal intestine, and this area could be more informative. However, that area presents difficulties of accession without invasive techniques and L-cells express GP43 and GP41 both in the small intestine and colon. Moreover, the targeted sequencing of the 16S ribosomal RNA gene has limitations in determining specific species and strains. Additionally, although a PICRUSt analysis has been done, little information on particular bacterial genes and their functions is obtained.

## 5. Conclusions

We have shown that a conventional eradication treatment in humans could improve carbohydrate metabolism by increasing GLP-1 secretion and that this change is closely related to alterations in the intestinal microbiota. Particular bacteria such as *Lachnobacterium*, *Bifidobacterium adolescentis*, and *Coriobacteriaceae* showed a possible association with GLP-1 secretion that deserves further investigation to decipher the possible mechanisms. Taken together, our findings reveal that, despite the decrease in microbiota biodiversity, a positive effect on GLP-1 secretion and on carbohydrate metabolism was observed. These results suggest that changes in the microbiota may exert metabolic effects that depend more on the equilibrium between the different species than on their biodiversity. Nevertheless, the impact of antibiotic use on metabolic profiles in humans requires further research.

## Figures and Tables

**Table 1:** Anthropometric and analytical characteristics of the study groups.

	Pre-*H. pylori* Eradication	Post-*H. pylori* Eradication	Control Group
**VARIABLES**	Mean ± SEM	Mean ± SEM	Mean ± SEM
**Sex** (female/male)	(24/16)	*__*	(12/8)
**Age** (years)	47.0 ± 2.0	__	43.6 ± 2.69
**Previous treatment with Omeprazole** (%)	35	*__*	20
**Anthropometry**			
Weight (kg)	72.8 ± 2.0	72.6 ± 2.0	72.5 ± 3.3
BMI (Kg/m^2^)	26.9 ± 0.7	26.2 ± 0.7	25.8 ± 1.0
Waist (cm)	92.3 ± 1.9	91.3 ± 1.9	89.3 ± 3.0
Hip (cm)	102.0 ± 1.3	102.9 ± 1.7	101.9 ± 2.3
**Blood pressure**			
Systolic (mmHg)	123.9 ± 2.6	125.4 ± 3.5	120.3 ± 3.0
Diastolic (mmHg)	77.8 ± 1.5	80.5 ± 1.8	75.6 ± 2.3
**Lipid Profile**			
TGs (mg/dL)	97.2 ± 6.3	93.5 ± 5.9	89.7 ± 9.3
TC (mg/dL)	194.2 ± 6.5	191.3 ± 6.3	177.1 ± 8.8
LDL-c (mg/dL)	121.5 ± 5.7 ^b^	118.0 ± 5.2	102.1 ± 7.6 ^b^
HDL-c (mg/dL)	53.0 ± 2.0 ^a^	55.4 ± 2.7 ^a^	57.0 ± 3.5
**Inflammation**			
CRP (mg/dL)	4.1 ± 0.4	3.6 ± 0.3	2.2 ± 0.7
**Glycemic status**			
HbA1c (%)	5.5 ± 0.08 ^a^	5.29 ± 0.06 ^a^	5.3 ± 0.1

BMI: body mass index, TGs: triglycerides, TC: total cholesterol, LDL-c: low-density lipoprotein cholesterol, HDL-c: high-density lipoprotein cholesterol, CRP: C-reactive protein, HbA1c: glycated hemoglobin. All values are means ± standard error of the mean. Student’s *t*-test for paired samples or the Wilcoxon test was used to compare the group of *H. pylori*-infected patients before and after antibiotic treatment. Student’s *t*-test or Mann–Whitney U tests were used between independent groups. Equal letter means differences between groups. a: differences between *H. pylori*-infected individuals before and after antibiotic treatment with *p* < 0.05. b: differences between *H. pylori*-infected patients and control group, *p* < 0.05.

**Table 2:** Simple linear correlation: differential microbial community and GLP-1 changes.

PHYLA	FAMILY/GENUS/SPECIES	% Change AUC GLP-1	% Change GLP-1 60’
Actinobacteria differential	*Coriobacteriaceae*	NS	r = 0.372 * p = 0.026
	*Bifidobacterium adolescentis*	r = 0.354 * p = 0.034	NS
Firmicutes differential	*Lachnobacterium*	r = −0.332 * p = 0.048	NS

* Correlation is significant at the 0.05 level. Spearman correlation test was used to compare the microbial differential abundance respect to GLP-1 changes in *H. pylori*-infected patients after antibiotic treatment. NS: non-significant differences.

## Supplementary Materials

The following are available online at https://www.mdpi.com/2077-0383/8/4/451/s1, Table S1: Simple linear correlation: microbial community and GLP-1 levels: Control group; Table S2: Simple linear correlation: microbial community and GLP-1: *H. pylori* positive (antibiotic treatment); Table S3: Simple linear correlation: microbial community and GLP-1: *H. pylori*-infected patients after antibiotic treatment; Figure S1: Relative abundances of the OTUs found significant with the correlations between changes in GLP-1 levels at minute 60 and the AUC for GLP-1 with percentage change in microbial community after antibiotic treatment.
