# Supplementary material for: H. pylori Eradication Treatment Alters Gut Microbiota and GLP-1 Secretion in Humans
Source: J Clin Med. 2019 Apr 4;8(4):451. doi: 10.3390/jcm8040451 (PMC6517938; doi:10.3390/jcm8040451)

Journal of
Clinical Medicine

MDPI

SUPPLEMENTARY TABLES:

Table S1. Simple linear correlation: microbial community and GLP-1 levels: Control group

PHYLA	FAMILY/GENUS/ SPECIES	GLP-1 baseline	GLP-1 30´	GLP-1 60´	GLP-1 120´	AUC GLP-1
Actinobacteria	*Micrococcaceae*	r=0.515* p=0.020	NS	NS	NS	NS
	Rothia	r=0.515* p=0.020	NS	NS	NS	NS
Bacteroidetes	*Bacteroidetes*	NS	NS	r=-0.456* p=0.043	NS	NS
	Bacteroidaceae	NS	NS	r=-0.512* p=0.021	NS	NS
	Bacteroides	NS	NS	r=0.350* p=0.021	NS	NS
Firmicutes	*Firmicutes*	NS	NS	r=0.457* p=0.043	NS	NS
	Peptococcaceae	NS	r=0.449* p=0.047	r=0.514* p=0.020	NS	NS
	Ruminococcaceae	NS	NS	r=0.621** p=0.004	r=0.475* p=0.034	r=0.474* p=0.035
	Megamonas	r=0.509* p=0.022	r=0.582** p=0.007	r=0.580** p=0.007	r=0.453** p=0.004	r=0.570** p=0.009
	Oribacterium	r=0.460* p=0.041	NS	NS	NS	NS

** Correlation is significant at the 0.001 level; *Correlation is significant at the 0.05 level.

Spearman correlation test was used to compare the microbial abundance respect to GLP-1 levels in control group.

Table S2. Simple linear correlation: microbial community and GLP-1: H. pylori positive (antibiotic treatment).

PHYLA	FAMILY/GENUS/ SPECIES	GLP-1 baseline	GLP-1 30´	GLP-1 60´	GLP-1 120´	AUC GLP-1
**Actinobacteria**	*Bifidobacterium. adolescentis*	**r=0.336* p=0.034**	**r=0.332* p=0.037**	NS	NS	**r=0.329* p=0.041**
**Bacteroidetes**	*Dysgonomonas*	NS	NS	**r=-0.321* p=0.042**	NS	NS
**Firmicutes**	*Turicibacteraceae*	NS	**r=0.358* p=0.023**	NS	NS	NS
	Turicibacter	NS	**r=0.358* p=0.023**	NS	NS	NS
	Streptococcus anginosus	NS	NS	**r=0.359* p=0.023**	**r=0.338* p=0.035**	NS
	Acidaminococcus	NS	NS	NS	**r=0.453** p=0.004**	**r=0.329* p=0.041**
	Blautia producta	**r=-0.439** p=0.005**	**r=-0.427** p=0.006**	**r=-0.330* p=0.038**	NS	**r=-0.387* p=0.015**
**Proteobacteria**	*Oxalobacteraceae*	NS	NS	**r=0.365* p=0.021**	NS	**r=0.327* p=0.042**
	Oxalobacter	NS	NS	**r=0.365* p=0.021**	NS	**r=0.328* p=0.042**
	O. formigenes	NS	NS	**r=0.365* p=0.021**	NS	**r=0.328* p=0.042**

** Correlation is significant at the 0.001 level; *Correlation is significant at the 0.05 level.

Spearman correlation test was used to compare the microbial abundance respect to GLP-1 levels in H. pylori-infected patients before antibiotic treatment.

Table S3. Simple linear correlation: microbial community and GLP-1: H. pylori-infected patients after antibiotic treatment.

FILOS	FAMILY/GENUS/E SPECIES	GLP-1 basal	GLP-1 30´	GLP-1 60´	GLP-1 120´	AUC GLP-1
Actinobacteria	*Actinobacteria*	**r=0,462** p=0,005**	**r=0,496** p=0,002**	**r=0,407* p=0,015**	**r=0,368* p=0,030**	**r=0,456** p=0,006**
	Bifidobacteriaceae	**r=0,343* p=0,038**	NS	**r=0,374* p=0,022**	NS	**r=0,357* p=0,030**
	Bifidobacterium	**r=0,369* p=0,029**	NS	NS	NS	NS
	B. longum	**r=0,442** p=0,008**	**r=0,414* p=0,013**	**r=0,434** p=0,009**	**r=0,391* p=0,020**	**r=0,450** p=0,007**
Bacteroidetes	*Prevotella*	**r=-0,396* p=0,017**	NS	**r=-0,340* p=0,042**	**r=-0,375* p=0,024**	**r=-0,369* p=0,027**
Firmicutes	*Ruminococcaceae*	NS	**r=0,341* p=0,045**	NS	NS	NS

** Correlation is significant at the 0.001 level; *Correlation is significant at the 0.05 level.

Spearman correlation test was used to compare the microbial abundance respect to GLP-1 levels in H. pylori-infected patients after antibiotic treatment.

Figure S1. Relative abundances of the OTUs found significant with the correlations between changes in GLP-1 levels at minute 60 and the AUC for GLP-1 with percentage change in microbial community after antibiotic treatment.

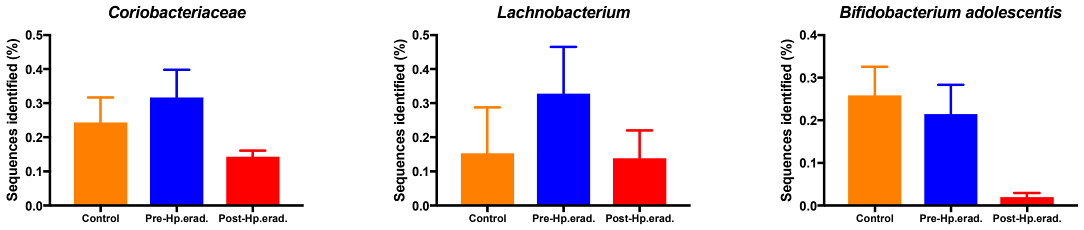

Supplement: Supplementary file 1 [file jcm-08-00451-s001.pdf]
